# Infection Complications after Lymphodepletion and Dosing of Chimeric Antigen Receptor T (CAR-T) Cell Therapy in Patients with Relapsed/Refractory Acute Lymphoblastic Leukemia or B Cell Non-Hodgkin Lymphoma

**DOI:** 10.3390/cancers13071684

**Published:** 2021-04-02

**Authors:** Felix Korell, Maria-Luisa Schubert, Tim Sauer, Anita Schmitt, Patrick Derigs, Tim Frederik Weber, Paul Schnitzler, Carsten Müller-Tidow, Peter Dreger, Michael Schmitt

**Affiliations:** 1 Department of Internal Medicine V—Hematology, Oncology & Rheumatology, University Hospital Heidelberg, 69120 Heidelberg, Germany; maria-luisa.schubert@med.uni-heidelberg.de (M.-L.S.); Tim.Sauer@med.uni-heidelberg.de (T.S.); Anita.Schmitt@med.uni-heidelberg.de (A.S.); Patrick.Derigs@med.uni-heidelberg.de (P.D.); carsten.mueller-tidow@med.uni-heidelberg.de (C.M.-T.); Peter.Dreger@med.uni-heidelberg.de (P.D.); michael.schmitt@med.uni-heidelberg.de (M.S.); 2 Department of Diagnostic and Interventional Radiology, University Hospital Heidelberg, 69120 Heidelberg, Germany; Tim.Weber@med.uni-heidelberg.de; 3 Department of Virology, University Hospital Heidelberg, 69120 Heidelberg, Germany; paul.schnitzler@med.uni-heidelberg.de

**Keywords:** CAR-T cell, infection, lymphodepletion, cytokine release syndrome

## Abstract

**Simple Summary:**

Chimeric antigen receptor T (CAR-T) cells have become clinical practice for the treatment of acute lymphoblastic leukemia and non-Hodgkin lymphoma. The aim of this retrospective study was to assess infection complications after lymphodepletion and CAR-T cell therapy. Infections were commonly detected, but manageable in most cases. Fast and appropriate identification as well as treatment were critical, especially in this very vulnerable patient group. Effective strategies to prevent infections as well as adequate medical management also include standardized prophylaxis and additional supportive therapy.

**Abstract:**

Chimeric antigen receptor T (CAR-T) cell therapy has proven to be very effective in patients with relapsed/refractory acute lymphoblastic leukemia (ALL) and non-Hodgkin lymphoma (NHL). However, infections—related either due to lymphodepletion or the CAR-T cell therapy itself—can result in severe and potentially life-threatening complications, while side effects such as cytokine release syndrome (CRS) might complicate differential diagnosis. Sixty-seven dosings of CAR-T cells in sixty adult patients with NHL (85%) and ALL (15%) receiving CAR-T cell therapy were assessed for infectious complications. Almost two-thirds of patients (61%) developed fever following lymphodepletion and CAR-T cell dosing. Microbiological or radiological findings were observed in 25% of all cases (bacterial 12%, viral 5%, fungal 8%). Inpatient infections were associated with more lines of therapy and more severe CRS. However, overall serious complications were rare after CAR-T therapy, with one patient dying of infection. Pathogen detection after inpatient stay was infrequent and mostly occurred in the first 90 days after dosing. Infections in CAR-T cell treated patents are common. Fast and suitable identification and treatment are crucial in these heavily pretreated and immunocompromised patients. In most cases infectious complications are manageable. Nonetheless, standardized anti-infective prophylaxis and supportive therapy are mandatory to reduce morbidity and mortality in CAR-T cell therapy.

## 1. Introduction

For patients with relapsed or refractory (r/r) B-lineage acute lymphoblastic leukemia (ALL) or r/r B-cell non-Hodgkin lymphoma (NHL), chimeric antigen receptor T (CAR-T) cell therapy has shown clinical efficacy [1,2,3]. In Europe, two CAR-T cell products are commercially available—axicabtagene ciloleucel (Axi-Cel; Yescarta^®^) and tisagenlecleucel (Tisa-Cel; Kymriah^®^). Axi-Cel is EMA-approved for r/r primary mediastinal B cell lymphoma (PMBCL) and diffuse large B cell lymphoma (DLBCL), while Tisa-Cel is approved for r/r DLBCL as well as r/r B-ALL in children and young adolescents (≤25 years).

Patients undergoing CAR-T cell therapy are at risk of bacterial, viral and fungal infections. Besides the underlying disease compromising immune response in affected patients, more than four prior chemotherapeutic treatment regimens, use of lymphodepleting chemotherapy, a higher dose of administered CAR-T cells and ALL as underlying disease are risk factors for infection after CAR-T cell treatment, as are cytopenia with a baseline absolute neutrophil count (ANC) below 500 cells/mm^3^ and b cell aplasia [4,5,6]. Moreover, treatment of CAR-T cell toxicities, i.e., the use of anti-cytokine treatment with tocilizumab or steroids, further compromises the immune response in respective patients [4,5] Due to similar symptoms of infections and cytokine release syndrome (CRS), differential diagnosis until pathogen detection can be difficult.

Here, we evaluated the frequency of infections in patients receiving CAR-T cell therapy during the initial hospitalization phase. Simultaneous occurrence of CRS and infectious disease (ID) as well as complications from infection were analyzed. Furthermore, we assessed infection in patients until six months after CAR-T cell therapy. The investigation period was divided into two parts: after discharge until day 90 (early follow-up) and day 90 until day 180 (late follow-up). Additionally, we also investigated patient immune status with B cell and white blood cell (WBC) counts, cumulative steroid dosage during hospitalization and CAR-T cell expansion as potential influencing factors for infection during both follow-up periods.

## 2. Materials and Methods

### 2.1. Patients

From September 2018 until September 2020, 60 patients at our center received CAR-T cells. Informed consent had been signed prior to treatment. Seven patients received two separate CAR-T cell dosings, resulting in a total of 67 dosings. Following CAR-T cell products being administered, the second-generation CAR-T cell products Axi-Cel comprising CD28 as costimulatory domain in a total dose of 0.4–2 × 10^8^ CAR-T cells, Tisa-Cel comprising 4-1BB (CD137) as costimulatory domain in a total dose of 0.6–6 × 10^8^ CAR-T cells and the third-generation CAR-T cell product (CD28 and 4-1BB costimulatory domains) were used within the clinical HD-CAR-1 trial at the Heidelberg University Hospital (EudraCT: 2016-004808-60; NCT: NCT03361748) [1,2,7]. 30 r/r DLBCL patients, two r/r PMBCL patients and one r/r mantle cell lymphoma (MCL) patient (as a compassionate use) received Axi-Cel. Tisa-Cel was administered to seven r/r DLBCL patients. The HD-CAR-1 product was administered in doses from 1 to 20 × 10^6^ CAR-T cells/m^2^ body surface area to four r/r DLBCL patients, three r/r MCL patients, two r/r follicular lymphoma (FL) patients, two r/r chronic lymphocytic leukemia (CLL) patients and nine ALL patients in an escalating dose form [7]. All patients received a lymphodepleting chemotherapy with fludarabine and cyclophosphamide prior to CAR-T cell administration. According to the Declaration of Helsinki, written informed consent for all patients was obtained. Ethical approval and approvals from the local and federal competent authorities were granted. HD-CAR-1 trial protocol received Institutional Review Board approval from the Ethics Committee (Medical Faculty of Heidelberg University, reference number: AFmu-405/2017) in October 2017. 

### 2.2. Anti-Infective Prophylaxis

Uniform evidence-based guidelines for prophylaxis before and after CAR-T cell therapy have not yet been established. Similar to patients receiving autologous or allogeneic stem cell transplantation, prophylaxis for herpes simplex (HSV) and varicella zoster (VZV), as well as prophylaxis for *pneumocystis jirovecii*, are widely recommended until recovery of a CD4+ T-cell count above 200/µL and/or up to one year after CAR-T cell dosing [8,9]. Prophylaxis for bacterial or fungal infection varies among CAR-T cell centers and there is currently no standard use in all CAR-T cell patients, although in neutropenic patients it is strongly recommended [8,10]. Anti-infective prophylaxis at the Heidelberg University Hospital is summarized in Table 1. 

#### Detection and Categorization of Infection

A standard-of-care (SOC) algorithm for fever after CAR-T cell treatment at the Heidelberg University Hospital included the following: after drawing blood cultures (from peripheral and, if available, central blood lines), appropriate antibiotic therapy was started (in neutrophil patients piperacillin/tazobactam was applied according to our standard operative procedure (SOP)). In cases of fever persistence or unclear fever beyond 48 h after antibiotic initiation, radiologic diagnostics, i.e., computed tomography (CT) of the chest, was carried out. Tests for pathogenic bacterial germs besides regular blood cultures included also other possible sources of infection such as catheter tips, which were also sent in for microbiological examination. 

Considering viral infections, panel testing in case of respiratory symptoms including screening for respiratory syncytial virus (RSV), influenza and parainfluenza virus and severe acute respiratory syndrome coronavirus 2 (SARS-CoV-2) was carried out. Furthermore, at regular intervals in case of immunosuppressive therapy (e.g., after allogeneic hematopoietic cell transplantation) in patient history, before initiating lymphodepletion or in case of suspicion during the hospitalization, the patient’s blood was tested for infections with hepatis B virus (HBV), hepatitis C virus (HCV), human immunodeficiency virus (HIV) as well as herpes virus species like herpes simplex virus (HSV), varicella zoster virus (VZV), Epstein-Barr virus (EBV) or cytomegalovirus (CMV). 

Fungal infections were diagnosed according to the 2008 revised European Organization for Research and Treatment of Cancer (EORTC) Consensus Group criteria including computed tomography (CT) of the chest to determine presence of typical imaging features, with proven, probable and possible invasive fungal disease [11].

### 2.3. Cytokine Release Syndrome Grading

CRS severity was graded according to consensus criteria [12].

### 2.4. CAR-T Cell Expansion 

Vector copy numbers of CAR-T cells for all used products (Axi-Cel, Tisa-Cel and HD-CAR-1) were assessed by single copy gene-based duplex-quantitative polymerase chain reaction (PCR) assay approach using standardized procedures [13,14].

### 2.5. Data Analysis 

Microsoft Excel^®^ was used for the statistical analysis and data collection. Data were analyzed by standard statistical measures, including median and range (minimum/maximum). IBM SPSS 20 for Windows (IBM Corp., Armonk, NY, USA) was used to calculate significance using the Students *T*-test. For all tests, 95%-confidence interval was calculated (denoted as CI) and *p*-values < 0.05 were considered statistically significant.

## 3. Results

### 3.1. Patients Characteristics

**Figure 1:**
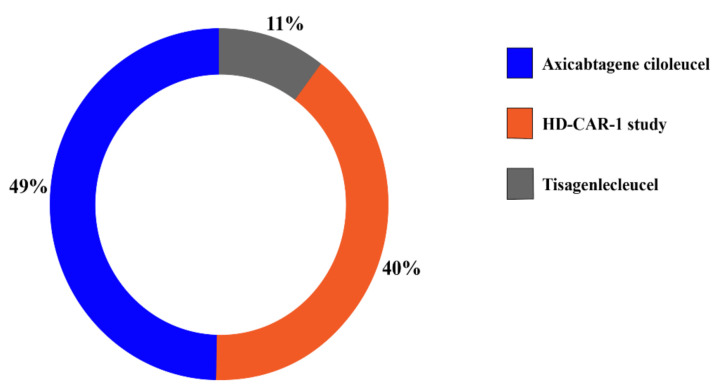
Distribution of CAR-T cell dosings by product. The main share of patients was treated with Axi-Cel (*n* = 33, 49%) and as part of the HD-CAR-1 study (*n* = 27, 40%). Seven patients received Tisa-Cel (11%).

Of the 60 patients included in this study, 40 were male and 20 were female; 51 patients (85%) were diagnosed with lymphoma and 9 patients (15%) with ALL. Of all lymphoma patients, 41 patients (69%) had diffuse large B cell lymphoma (DLBCL), 4 (7%) mantle cell lymphoma (MCL), 2 (3%) chronic lymphocytic leukemia (CLL), 2 (3%) follicular lymphoma (FL) and 2 (3%) patients had primary mediastinal B-cell lymphoma (PMBCL) (Table 2). 33 (49%) of 69 CAR-T dosings were performed with Axi-Cel, 27 (40%) with the HD-CAR-1 product and seven (11%) with Tisa-Cel (Figure 1). All seven patients receiving a second CAR-T cell dosing were HD-CAR-1 patients.

### 3.2. Baseline and Disease Analysis

Age (56 years, range 20–74), Body mass index (24.4, range 15.9–45.5) and Karnovsky index (90, range 50–100) of the patients receiving CAR-T cells by gender (male 66.6%) are summarized in Table 2. Patients had received a median of 5 prior therapy lines (range 2–10) and had a median hospitalization duration of 20 days (range 14–110) following CAR-T cell administration. Four patients died after CAR-T cell therapy, three due to progressive disease and one by infection with CMV.

### 3.3. Fever and Cytokine Release Syndrome (CRS)

Overall, after 41 CAR-T cell dosings (61.2%) fever occurred (Table 3). In 11.9% of cases (8 dosings) fever developed after administration of lymphodepleting therapy before CAR-T cell dosings. CRS was diagnosed after 33 (49.2%) CAR-T cell dosings. Grade I CRS (64 %/*n* = 21) and grade II CRS (30%/*n* = 10) were most common, whereas grade III and IV were rarely observed (3%/each *n* = 1).

### 3.4. Bacterial Infection and Antibiotic Treatment

**Figure 2:**
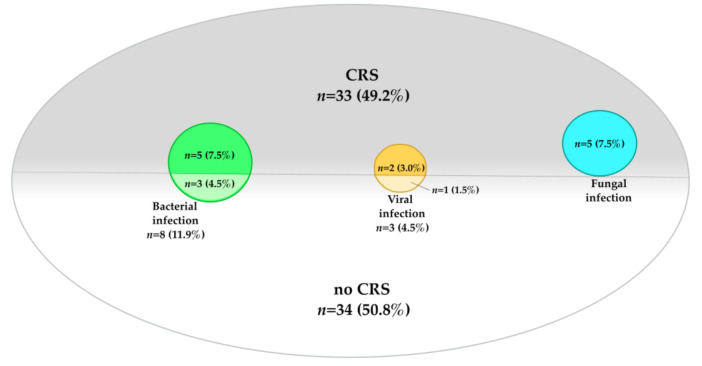
Distribution of infection and CRS. This graphic shows the distribution of infections—divided into bacterial (*n* = 8, 11.9%), viral (*n* = 3, 4.5%) and fungal (*n* = 5, 7.5%) origin and CRS (*n* = 33, 49.2%). Furthermore, infections with simultaneous CRS (*n* = 12, 17.9%) are displayed.

After eight dosings (11.9%), bacterial pathogens were identified (Table 3). Here, staphylococci (8.9%/*n* = 6) with subgroups *S. epidermidis* (7.5%/*n* = 5) and *S. haemolyticus* (1.4%/*n* = 1), *Klebsiella pneumoniae* and *Escherichia coli* (each 1.4%/*n* = 1) were detected. According to our on-house SOP, all patients who experienced fever received antibiotic therapy (61.2%/*n* = 41). Median duration of antibiotic treatment was 10 days (range 4–40). Of the aforementioned eight dosings with confirmed pathogens, five patients were simultaneously diagnosed with CRS (Figure 2).

### 3.5. Viral Infection and Antiviral Treatment

**Figure 3:**
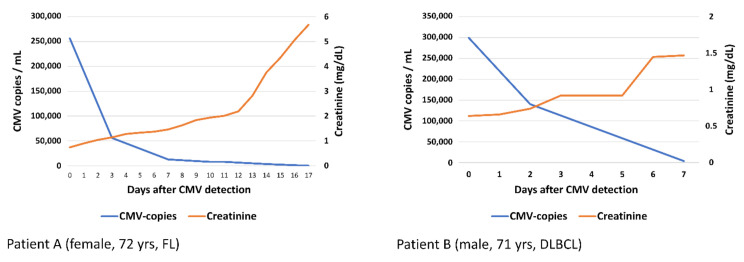
Laboratory values (CMV-DNA, measured by PCR, and creatinine). Laboratory values of CMV copies and creatinine of patient (**A**) and patient (**B**) are displayed. Both patients developed acute kidney failure, most likely as an adverse effect from foscarnet.

Viral infections were observed after three dosings (4.5%), all after the CAR-T cell had been administered (Table 3). Detected viral pathogens were CMV (2.9/*n* = 2) and RSV (1.4%/*n* = 1), with a median treatment (CMV: foscarnet; RSV: ribavirin) duration of 15 days (range 8–30). Two of the abovementioned patients (one diagnosed with reactivated CMV and one with detected RSV) developed CMV and RSV pneumonia, respectively (see case report in Section 3.8). Laboratory values of CMV copies and creatinine of both patients are displayed in Figure 3, as both developed acute kidney failure, most likely related to foscarnet treatment. In two patients with viral infections (2.9%), CRS was simultaneously diagnosed (Figure 2).

### 3.6. Fungal Infection and Antifungal Treatment

Five patients (7.5%) developed a suspected fungal infection after CAR-T cell dosing (Table 3). Anti-fungal treatment had a duration of 13 days (range 12–19). In four cases, radiological evaluations were suspected to be of fungal origin and classified as possible invasive fungal disease according to EORTC criteria. In the other patient, additional to radiological findings, aspergillus fumigatus in bronchoalveolar lavage (BAL) was detected (probable invasive fungal disease classification). Concomitant CRS was evident after all five dosings (7.5%) (Figure 2).

### 3.7. Overview of Infections and Complications

Overall, in 16 CAR-T cell dosings (23.8%) a pathogen or cause for infection was detected. Occurrence of bacterial, viral and fungal infections was equally distributed (bacterial/viral: 8 [CI 3–10] vs. 3 [CI 1–6], *p* = 0.1; bacterial/fungal: 8 [CI 3–10] vs. 5 [CI 3–9], *p* = 0.2; viral/fungal: 3 [CI 1–6] vs. 5 [CI 3–9], *p* = 0.2). Furthermore, a comparable duration of therapy was observed (bacterial/viral: 10 [CI 10–15] vs. 15 [CI 5–30] days, *p* = 0.2; bacterial/fungal: 10 [CI 10–15] vs. 13 [CI 10–19] days, *p* = 0.3; viral/fungal: 15 [CI 5–30] vs. 13 [CI 10–19] days, *p* = 0.3). DLBCL was the underlying disease in 12 of 16 cases, followed by FL and ALL (each 2). For the group of patients with infection, three patients developed sepsis (all with a sequential organ failure assessment (SOFA) score >8) and required catecholamine therapy with noradrenaline for circulatory support as well as invasive ventilation. In addition, post-infection kidney failure occurred in eight of these patients where infection had been confirmed. When compared to all other dosings without infection, all the abovementioned complications were found to be significantly more frequent in patients with infection (catecholamine usage: 3 [CI 3–5] vs. 2 [CI 2–3], *p* = 0.02; invasive ventilation: 3 [CI 2–4] vs. 1 [CI 0–1], *p* = 0.004; post-fever kidney failure: 8 [CI 6–10] vs. 1 [CI 0–3], *p* < 0.001). Catecholamine use and invasive ventilation occurred significantly more often in patients with fungal compared to those with bacterial infection (2 [CI 2–3] vs. 0 [CI 0–1], *p* = 0.03 each), whereas other parameters, i.e., age or gender, did not influence the rate of catecholamine use. Furthermore, infection was not associated with administered CAR-T cell dosage (*p* = 0.2). However, CAR-T cell dose was significantly correlated with a higher grade of CRS (median grades 2 [CI 2–4] vs. 0 [CI 0–1], *p* < 0.001) and a higher number of prior therapies (median 6 [CI 5–10] vs. 3 [CI 2–4], *p* = 0.01). 

### 3.8. Case Reports:

(a) CMV pneumonia: male patient, 71 years old, DLBCL, treated with Axi-Cel. 

**Figure 4:**
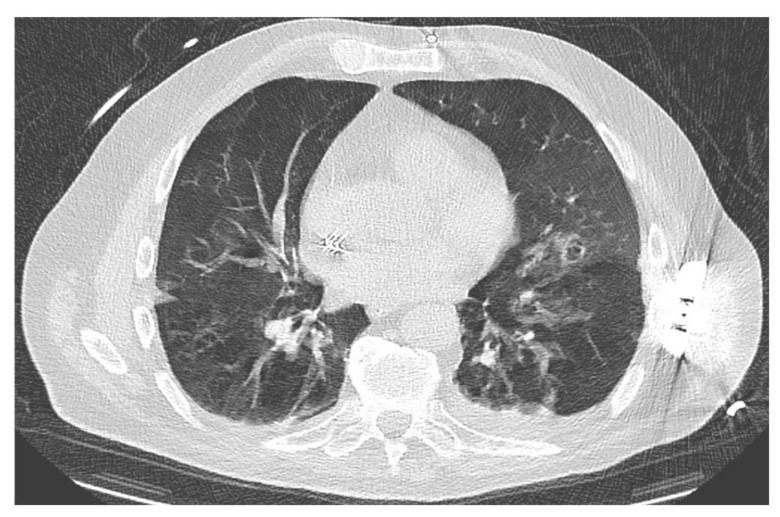
CMV pneumonia. CT scan with diffuse bronchocentric pulmonary ground-glass opacities predominantly in the left lower and left upper lobe.

On day 29 after administration of CAR-T cells (extended inpatient stay after severe neurotoxicity—ICANS IV), the patient developed fever and an increasing need for oxygen supplementation. After drawing of blood cultures and initiation of antibiotic treatment with piperacillin/tazobactam, a CT scan revealed diffuse bronchocentric pulmonary ground-glass opacities predominantly in the left lower and left upper lobe (Figure 4). In addition, examination of both serum and BAL specimens were positive for CMV, strongly indicating CMV reactivation. Due to pancytopenia, antiviral therapy with foscarnet was initiated. However, the patient died due to multiple organ failure induced by pneumogenic sepsis on day + 7 after CMV detection.

(b) RSV-B pneumonia: male patient, 68 years old, DLBCL, treated with Axi-Cel.

A chest CT scan (day 5 before CAR-T dosing) showed bronchocentric consolidation and ground-glass opacities in the right upper lobe as well as areas with centrilobular nodules and tree-in-bud pattern in both lungs. On the third day after receiving the lymphodepleting therapy, the patient developed an increase in inflammation markers and a body temperature raised up to 38.2 °C. With a rapidly developing and increasing cough, a second set of CT scans was carried out without evidence of an infiltrate. However, a panel examination for respiratory viruses as additional diagnostics confirmed RSV-B in the throat swab. According to our in-house SOP, a therapy with ribavirin was initiated. Ribavirin could be discontinued after 31 days of treatment following negative panel testing for RSV and the patient was released in good general health condition 50 days after receiving CAR-T cells.

### 3.9. Infection within the First 180 Days—Overall Occurance and Evaluation of Risk Factors

**Figure 5:**
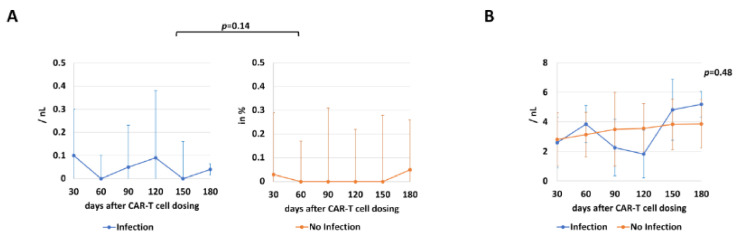
Immune status by measurement of B cell and WBC counts (median and CI). For assessment and comparison of immune status, B cell (**A**) and WBC (**B**) counts (per nanoliter; nL) of patients with and without infection after inpatient stay are shown at different timepoints ranging from day 30 to 180.

Patients were intensively monitored after discharge and medical records were obtained from 56 dosings (83.5%), with unavailability in the other 11 dosings either due to death or loss of follow-up. For early follow-up, infections were observed in four patients—three of bacterial (two patients with S. epidermidis and one with *E. coli*) and one of viral (parainfluenza) origin. Another infection was identified in the late follow-up period: here, radiological evaluations were suspected to be of fungal origin and were classified as possible invasive fungal disease according to EORTC criteria, leading to a total of five patients with post-discharge infections in the overall follow-up after CAR-T cell dosing. B cell and white blood cells were comparable in both infection and non-infection cohorts (*p* = 0.14 and *p* = 0.48, respectively) (Figure 5). 

**Figure 6:**
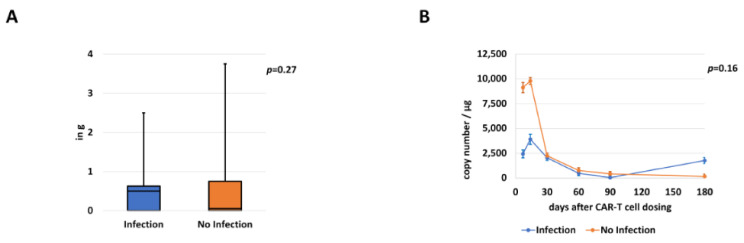
Comparison of cumulative steroid dosages and CAR-T cell expansion (median and CI). Comparison of steroid dosages (**A**) received during hospitalization in patients with or without infection (in gram; g). Additionally, CAR-T expansion levels (**B**) in both subgroups are displayed.

Cumulative steroid dosages (in cortisone equivalence dosage) were also assessed, showing no significant differences in patients with (median 0.5 g [CI 0.0–2.5]) and without (median 0.1 g [CI 0.0–3.8]; *p* = 0.27) infection (Figure 6A). In addition, we analyzed CAR-T cell expansion in both groups at different time points (day 7, day 14, day 30, day 60, day 90 and day 180). Here, copy numbers per µL were comparable over the investigated time period (*p* = 0.16). (Figure 6B).

## 4. Discussion

Infectious complications constitute a risk for hospitalized cancer patients, particularly those treated with lymphodepleting chemotherapy as conditioning before CAR-T cell therapy. In this study on 67 CAR-T dosings in 60 patients with r/r ALL and NHL, the risk of infection was associated with the extent of prior treatment and the severity of CRS. Requirement of vasopressors and invasive ventilation was more frequent in patients with infection. However, overall lethal complications were rare. 

In prior studies, infectious complications after CAR-T cell dosing were common, with bacterial, viral and fungal pathogens being the most frequent causes [15,16,17,18]. Particularly, infections with bacterial pathogens were shown to be frequent, occurring in about 15–20% of cases in previous studies on patients who had received CAR-T cell therapy [5,16]. The spectrum of pathogens has been described as very broad, with both gram-positive and negative, anaerobic and mycoplasm being detected. Neutropenic patients generally have a different pathogen environment than immunocompetent patients [19]. Our findings are comparable to the abovementioned studies. Of the 35 dosings with fever, only in eight patients a bacterial strain could be isolated (22.8%), similar to evaluations in other studies in patients with leukopenia (10 to 25%) [19,20]. Oftentimes, these arose from the patients’ endogenous flora [21]. Of the pathogens detected, staphylococcus species (with *S. epidermidis* and *S. haemolyticus* being known endogenous skin flora) was found in 75%. 

Most viral infections in patients after CAR-T cell therapy have been associated with respiratory viruses such as RSV, rhinoviruses, influenza and parainfluenza virus [16]. In some cases, viral infection is a reactivation of previous viral infections. Causes described in studies include HBV, HSV, VZV, EBV or CMV [12,15,22]. In our cohort, infections with CMV and RSV were detected, causing severe pneumonia in two cases. Both patients suffering from CMV were negative in screen testing before lymphodepletion chemotherapy and in additional regular testing for CM-viremia during their respective hospitalization. This illustrates the difficulties regarding early detection of virus reactivation. Therefore, regular tests should be performed at fixed intervals.

Fungal pathogens were also identified as probable cause for infection after lymphodepletion and CAR-T cell administration in several studies. Frequently detected pathogens were *Aspergillus*, *Candida*, *Fusarium* or *Coccidioides* spp. [18,23]. In one patient in our cohort, *Aspergillus fumigatus* was detected in the BAL fluid, while all other four patients had possible invasive fungal disease diagnosed by CT imaging.

Overall, most infections occurred early after CAR–T cell infusion during hospitalization or in the first 90 days after dosing. In our study, B cell and WBC counts were not associated with higher risk for infection in patient follow-up, while steroid dosages and CAR-T cell expansion were also shown to be comparable in both groups. However, copy numbers were higher in early measurements on days 7 and 14 in patients without infection, warranting further investigation with more timepoints and in bigger cohorts.

Differential diagnosis of fever in patients receiving CAR-T cells is complex. Firstly, the patients might develop fever due to different causes such as the abovementioned bacterial, viral or fungal pathogens. As differential diagnosis, an incipient CRS must always be taken into account, which is statistically more frequent than a pathogen detection. High-grade CRS (grade ≥ 3) itself has been described as a risk factor for infection [4,5,15,24,25]. On the other hand, earlier studies as well as our evaluation underline the need for prophylaxis. These have shown to prevent infections as well as mitigate possible serious disease courses. In addition, adequate and rapid treatment of infections as a “hit hard and early” approach is important. This can reduce serious complications and the risk of fatal courses, especially in the immunocompromised host. 

All of these evaluations show the need for special precautions and planning for patient follow-up care [26,27]. After administration of CAR-T cells, extensive monitoring is necessary until complete immune reconstitution. In this respect, follow-up of CAR-T cell patients should be performed in analogy of follow-up strategies for patients after allogeneic hematopoietic cell transplantation (alloHCT). Both patient groups share similarities regarding their immuno-reconstitution as well as potential complications. Because of this, regular follow-up testing should include testing, for example, of virus copies for CMV and EBV in order to detect reactivations at an early stage and treat them promptly before possible complications occur. In addition, this follow-up should also include other measures such as vaccination, e.g., for seasonal influenza or *pneumococcus* spp., as well as booster vaccinations for low titers. In the case of recurrent pneumonia, early control CT scans and early use of BAL should be considered. Moreover, this report as well as other evaluations of potential infections in patients receiving CAR-T cells underline the importance of standardized approaches to antimicrobial prophylaxis regimens in CAR–T cell recipients as well as special supportive care guidelines [8,10,24]. The approach in this study, with CAR-T dosing according to our SOP providing infection prevention with antibiotic, -viral and -fungal prophylaxis as well as “hit hard and early” anti-infective therapy in case of fever or other signs of infection, proved effective. However, with rates of prophylaxis before and after CAR-T cell therapy (Table 4) of only 13% antibiotic, 55% antiviral and 61% antifungal prophylaxis CAR-T cell dosing (61%, 88% and 90% afterwards, respectively), this leaves further room for improvement. Future goals include the identification of additional risk factors for infection and its timeframes as well as assessment of influencing factors in the pathogenesis of infection such as gut microbiota [28]. Moreover, evaluation of infections occurring before lymphodepletion and those after the infusion of CAR-T cells is necessary because of potential connections between the two. This further analysis could provide additional development and strengthening and improvement of infection prevention systems, as well as for safer therapy delivery and better patient outcomes. 

This study included CAR–T cell recipients with six disease types as well as three different CAR-T cell products, standardized supportive-care measures and infection monitoring for the full duration of hospitalization. Also included was a full analyses of preexisting baseline and post-CAR–T cell infusion risk factors for infection with a follow-up of 180 days.

Limitations include missing evaluation of infection beyond the follow-up period of 180 days. The results obtained are subject to limitations as a retrospective analysis, and with 67 CAR-T cell dosings, there was a small number of examined patients and infection events, both for hospitalization and follow-up, compared to the approval studies. All the findings of this analysis therefore need to be further evaluated and confirmed in randomized prospective studies or in a large meta-analyses.

## 5. Conclusions

While infectious complications commonly occur after lymphodepletion and CAR-T cell dosing, incidence and type of infections were consistent with those seen in patients treated for other r/r B-cell malignancies. Overall, infections (24%) were manageable, when supported by fast identification and initiation of treatment. Fatal infections were rarely observed (1.5%), especially among patients receiving optimized lymphodepletion chemotherapy and CAR–T cell dosing regimens. Standardized strategies to reduce risk and prevent infections in immunocompromised patients are pivotal for successful CAR-T cell dosing and should therefore be firmly established analogous to other hematological therapies such as alloHCT.

## Figures and Tables

**Table 1:** Recommended prophylaxis at Heidelberg University Hospital.

Antibiotic	Oral: rifaximin 200 mg 2 × 1 until neutrophil regeneration
Antiviral	Oral: aciclovir 2 × 400 mg daily until CD4 regeneration Intravenous: aciclovir 2 × 5 mg/kg daily
Antifungal	Oral: co-trimoxazole 960 mg 1 × 1 only Mon, Wed, Fri for PCP prophylaxis (from day 0, accompanying folic acid 5 mg 2×/week) up to CD4 regeneration; fluconazole 200 mg, 0-1-0 until neutrophil regeneration (or if there is anamnestic evidence of fungal pneumonia: posaconazole: day 1: 100 mg 3-0-3; from day 2: 300 mg 0-1-0) Intravenous: co-trimoxazole 960 mg Mon, Wed, Fri for PCP prophylaxis (from day 0, accompanying folic acid 5 mg 2×/week until regeneration); fluconazole 200 mg 0-2-0

The dosages should be adjusted to the current kidney function. Alternative preparations should be used in the event of intolerance. PCP = *pneumocystis pneumonia* (also known as PJP for *pneumocystis jiroveci* pneumonia).

**Table 2:** Baseline and disease characteristics.

Baseline Characteristics	All Patients *n* = 60	Male *n* = 40	Female *n* = 20
Age (years), median (range)	56 (20–74)	57 (20–71)	47 (20–74)
Body mass index, median (range)	24.4 (15.9–45.5)	26.4 (18.7–45.5)	21.7 (15.9–41.1)
Karnofsky index (percent), median (range)	90 (50–100)	90 (50–100)	90 (50–100)
Disease characteristics	All patients *n* = 60		
Disease Prior therapy lines median (range)	DLBCL (69%), ALL (15%), MCL (7%), CLL (3%), FL (3%), PMBCL (3%) 5 (2–10)		
Duration in-patient stay (days), median (range)	20 (14–110)		

DLBCL = Diffuse large B-cell lymphoma, ALL = Acute lymphoblastic leukemia, MCL = Mantle cell lymphoma, CLL = chronic lymphocytic leukemia, FL = Follicular lymphoma, PMBCL = Primary mediastinal B-cell lymphoma.

**Table 3:** Microbiological characteristics and events.

Events	Occurrence		
Fever (in %/*n*)	61.2/41		
Cytokine release syndrome (in %/*n*)	49.2/33: grade I 64%/21, grade II 30%/10, grade III 3% & grade IV 3%/each 1		
	Bacterial infection	Viral infection	Fungal infection
Treatment * (in %/*n*)	61.2/41	4.5/3	7.5/5
days of application median (range)	10 (4–40)	15 (8–30)	13 (12–19)
Cultured pathogen detected/suspected radiological diagnosis (in %, *n*)	11.9/8	4.5/3	7.5/5
simultaneous CRS (in %, *n*)	7.5/5	3.0/2	7.5/5

* Either antibiotic/antiviral/antifungal, no prophylactic applications.

**Table 4:** Anti-infective prophylaxis before and after hospitalization.

	Prior Admission (in %, *n*)	After Discharge (in %, *n*) †
Antibacterial prophylaxis *	13.4/9	60.9/40
Antiviral prophylaxis **	55.2/37	88.0/59
Antifungal prophylaxis	C 61.1/41, F 17.9/12, P 2.9/2, V 2.9/2	C 89.5/60, F 62.6/42, P 4.4/3, V 4.4/3

* = Either rifaximin, ciprofloxacin or amoxicillin/clavulanic acid; ** = acyclovir, † = in 5.9% of applications patient deceased during stationary stay and therefore received no further medication; C = co-trimoxazole, F = fluconazole, P = posaconazole: V = voriconazole (if both: C and either F/P/V).

## Data Availability

The data presented in this study are available in this article.
